# Supplementary material for: The interactome of intact mitochondria by cross-linking mass spectrometry provides evidence for coexisting respiratory supercomplexes
Source: Mol Cell Proteomics. 2017 Dec 8;17(2):216–32. doi: 10.1074/mcp.RA117.000470 (PMC5795388; doi:10.1074/mcp.RA117.000470)
Supplement: Supplemental Data [file supp_RA117.000470_133922_0_supp_23978_fzffwf.zip › spectra_annotation/mito_DR_spectra_annotation/494-1-9-1-7-1.pdf]

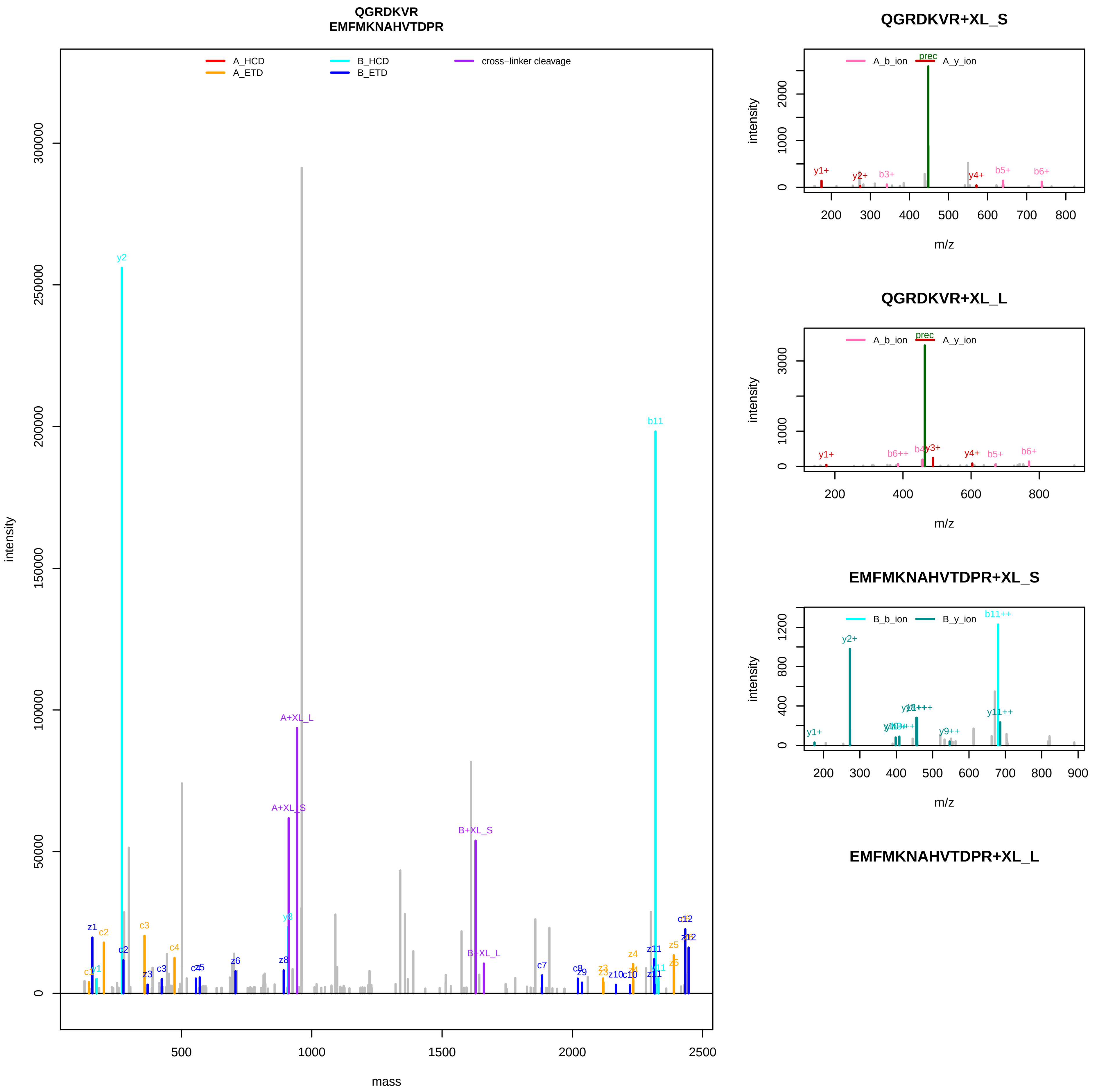
QGRDKVR
EMFMKNAHVTDPR
A_HCD
A_ETD
B_HCD
B_ETD
cross−linker cleavage
intensity
mass
y2
b11
A+XL_L
A+XL_S
B+XL_S
B+XL_L
y8
z1
c2
c3
c4
z3
z6
z8
c7
c9
z9
z10
c10
z4
z11
z5
c12
z12
QGRDKVR+XL_S
A_b_ion
A_y_ion
prec
y1+
y2+
b3+
y4+
b5+
b6+
m/z
QGRDKVR+XL_L
b6++
y3+
EMFMKNAHVTDPR+XL_S
B_b_ion
B_y_ion
b11++
y2+
y9++
y11++
EMFMKNAHVTDPR+XL_L
